# Pleomorphic adenoma presenting as retroauricular mass: an unusual case

**DOI:** 10.11604/pamj.2020.37.209.22059

**Published:** 2020-11-02

**Authors:** Laina Ndapewa Angula, Le Sun, Ning Fang, Xin Wang

**Affiliations:** 1 Department of Otolaryngology, First Hospital of Jilin University, Changchun, China

**Keywords:** Pleomorphic adenoma, parotid gland, retroauricular mass

## Abstract

Pleomorphic adenomas are benign salivary gland tumours that mostly affect the superficial lobe of the parotid gland. They are commonly found incidentally as rigid, unilateral, painless masses, growing gradually. Ninety percent of pleomorphic adenomas occur in the parotid gland, while 10% appear in minor salivary glands. The incidence of the parotid tumour is 2.4 in 10,000 per year. We describe the case of a 53-year-old woman presenting with a gradually growing retroauricular mass on the left side of the ear. Radiographic imaging and histopathological findings suggested a pleomorphic adenoma of the parotid gland. The tumour was removed and the patient recovered well upon follow-up.

## Introduction

Pleomorphic adenoma is well recognized as a benign mixed tumour. It is the most frequently encountered salivary tumour in the clinic, accounting for two-thirds of all salivary gland neoplasms [1]. In general, pleomorphic adenomas occur in the parotid glands (85%), salivary glands (10%), and submandibular glands (5%) [2]. A broad diversity of histological benign and malignant neoplasms may appear in the parotid glands, due to the heterogeneous mix of cells and tissues. Pleomorphic adenoma commonly arises as a gradual, continuous, asymptomatic, inflammation of the parotid gland, with no facial nerve association [3]. The best recommended surgical approach for the parotid gland tumour is conservative parotidectomy [4]. Herein, we describe a case of pleomorphic adenoma of a parotid gland occurring in a 53-year-old woman patient.

## Patient and observation

**Figure 1:**
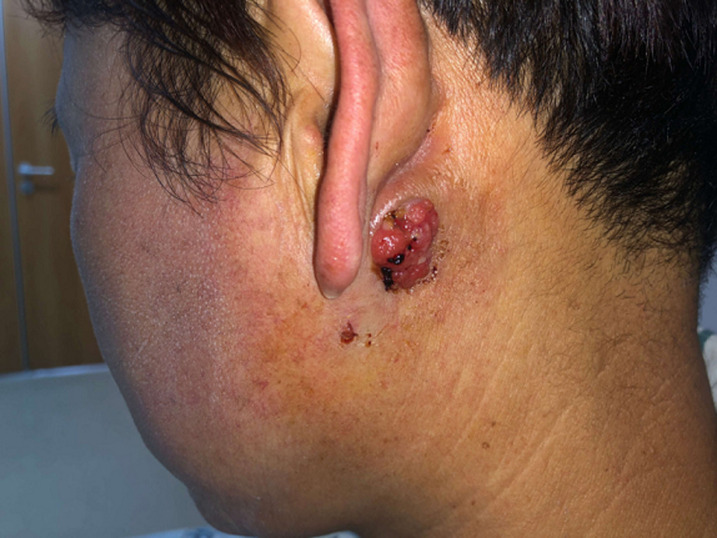
lateral view of the neck showing the left postauricular mass

**Figure 2:**
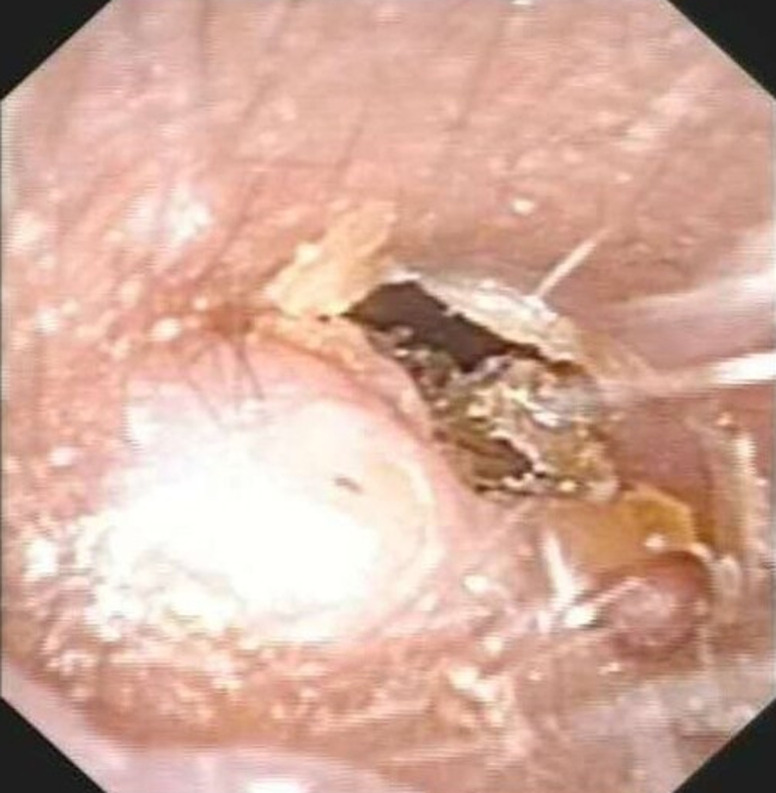
otoscopic findings of the posterior wall of the left auditory canal showing a mass; a normal tympanic membrane was seen

**Figure 3:**
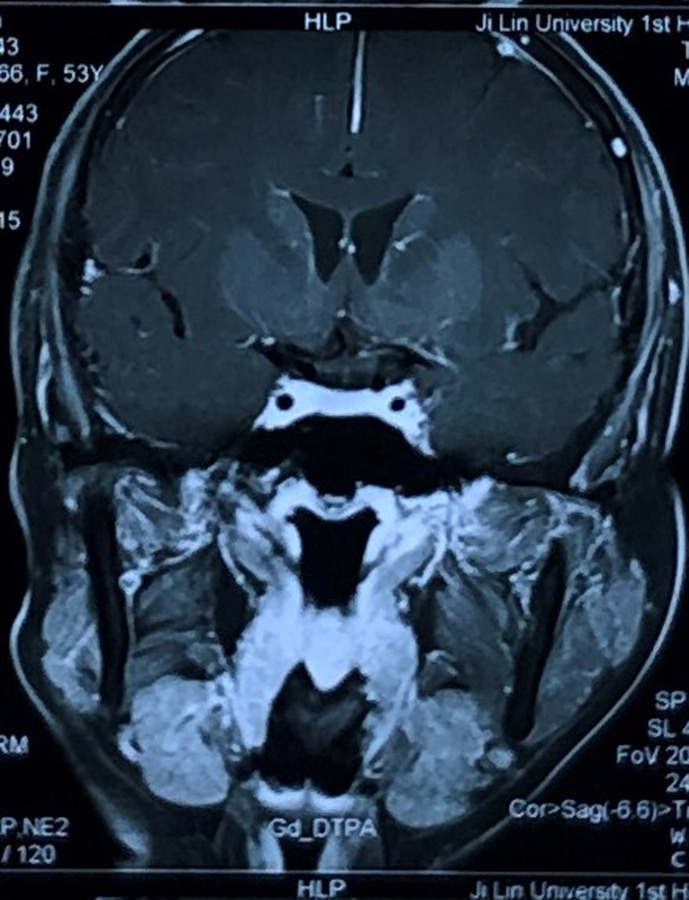
MRI displaying a hypointense lesion

**Figure 4:**
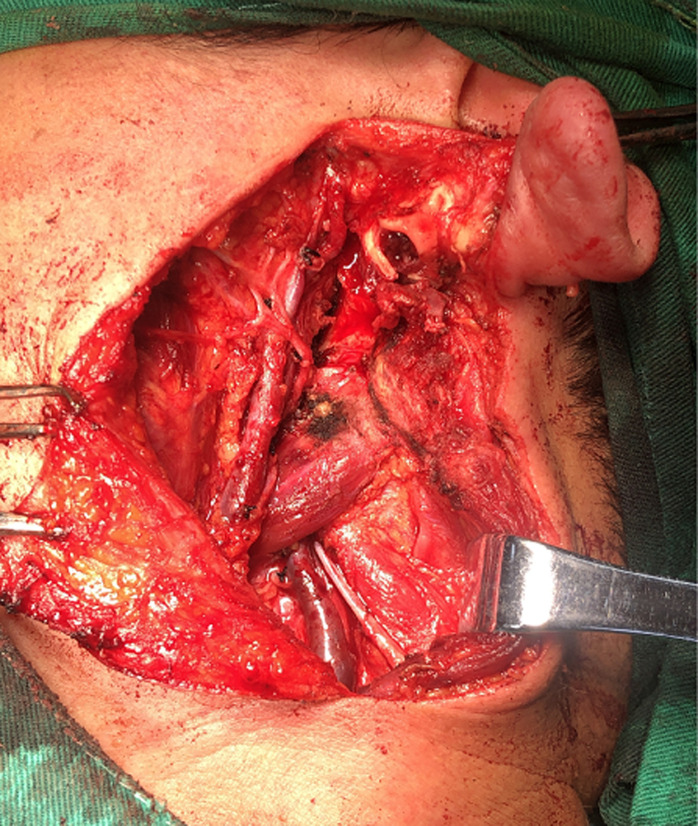
facial nerve trunk exposed and protected

**Figure 5:**
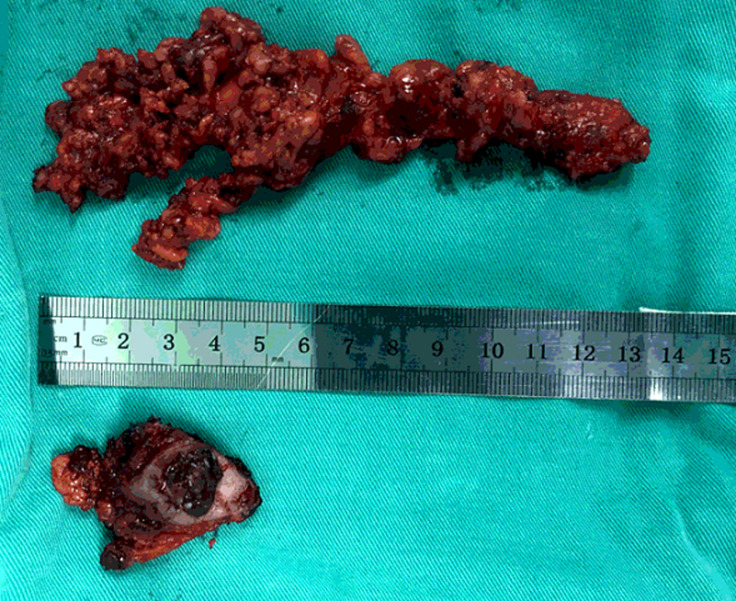
removed parotid gland, lymph nodes, and adipose connective tissue of size 2 cm × 2 cm × 1.4 cm

**Figure 6:**
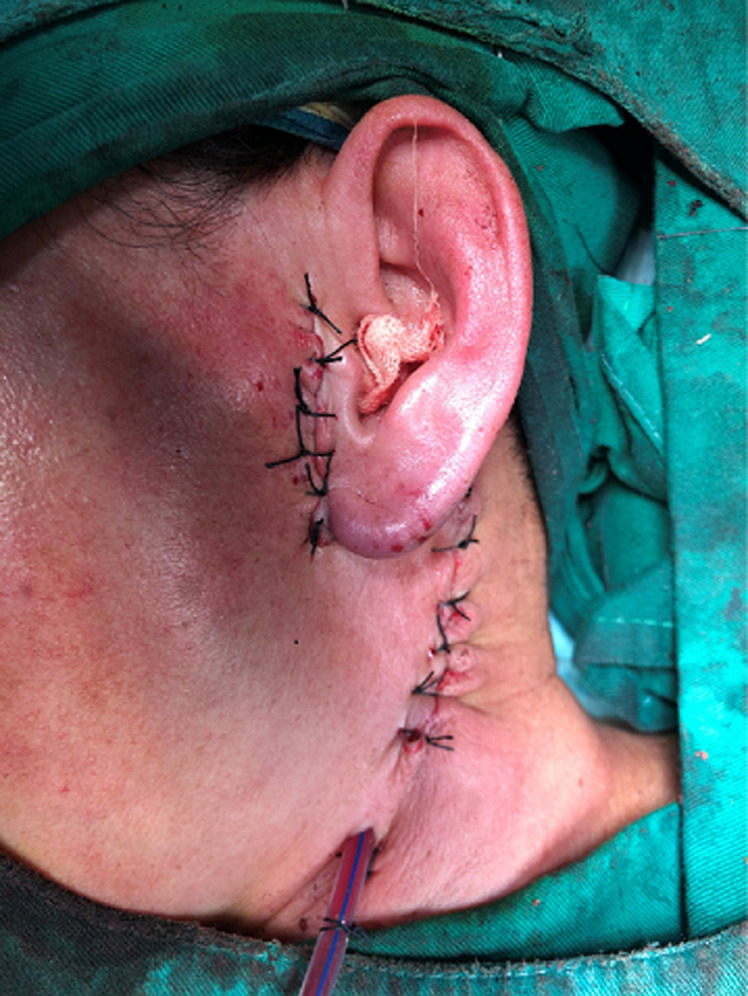
sutured layer with a drainage tube

**Figure 7:**
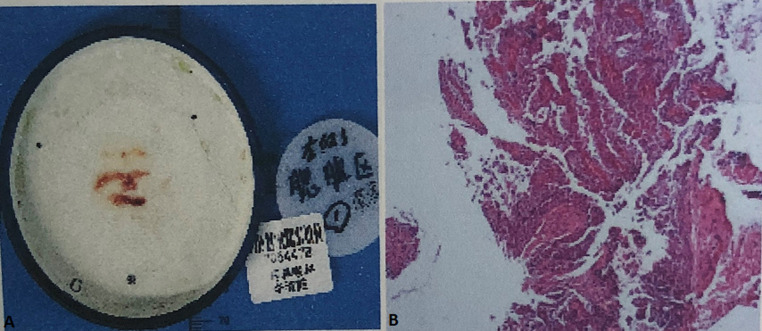
A,B) biopsy of the left parotid gland revealing a malignant tumour, a persistent mucoepidermoid carcinoma

A 53-year-old woman presented with a posterior left retroauricular mass, that was discovered 10 months ago and resected respectively at a local hospital. Ten days after this operation, a pruritic, painless lesion of a size 2 cm x 2 cm was observed at the incision area (Figure 1). The patient had no history of migraine, loss of hearing, drug allergy, or trauma. Otoscope examination revealed an external auditory meatus protrusion, with a normal tympanic membrane (Figure 2). Magnetic resonance imaging (MRI) revealed a heterogeneous lesion (Figure 3). The patient underwent extended left parotidectomy. An s-incision of 10 cm long was made extending from the earlobe towards the mandibular angle. With palpation, the tumour was located in the parotid gland. The superficial lobe of the parotid gland was gradually removed with caution to the facial nerve trunk (Figure 4). The tumour had invaded the deep lobe of the parotid gland and the posterior wall of the external auditory canal. The deep lobe of the parotid gland was removed simultaneously, and the left cervical lymph node dissection was performed in levels II-IV, with its adipose connective tissue. The diameter of the larger lymph node being 2 cm (Figure 5). A method of sleeve resection was used to remove the protruding posterior wall of the external auditory canal. A drainage tube was inserted (Figure 6). There was no complication, the operation was completed and the patient recovered well. The pathological results showed that the tumour of about 2 cm x 2 cm x 1.4 cm, involved the epidermis, vessels, and nerves. There was a distribution of the lymph node with metastasis and distribution of the external auditory canal with mucoepidermoid carcinoma. Immunohisto chemistry results showed CK7 (+), calponin (-), CK5/6 (+), P63 (+), S-100 (-), Dog-1 (+), CD117 (-), Ki-67 (+5%), P53 (-), and EMA (+). Hematoxylin and eosin (H&E) staining indicated a D-PAS positive (Figure 7). The clinical diagnosis was pleomorphic adenoma.

## Discussion

World Health Organization has described pleomorphic adenoma as clear-defined metastasis distinguished by its chondroid, mixed and mucoid appearance [5]. Patients´ ages range from 13 to 87 years, with an average of 46 years, and a limited predilection for women [6]. Usually, a pleomorphic adenoma originates at the head and neck region, as irregular in shape, not fixed and, gradually progressing [7]. Pleomorphic adenomas often recur, and in some cases even advances to malignant transformation. Extended parotidectomy is recommended for pleomorphic adenoma [8]. Simple tumour enucleation should be avoided, as it is known with an increased risk of violation of the pseudopodia and local tumour recurrences, therefore parotidectomy is more beneficial. Adult age at diagnosis, obesity, and exposure to radiation is associated with increased risk in major salivary gland cancer [9]. Facial movements are a good indication of facial nerve functions.

MRI is preferred for its exceptional illustration of soft tissue and its characterization of tumour border and relationship with enclosing structures [10]. The histopathological characteristics of this case suggested pleomorphic adenoma. A Ki-67 index is an important tool in differentiating pleomorphic adenoma from non-pleomorphic adenoma and identifying the extend of the malignant transformation [11]. P63 positive expression is highly related to various human cancers such as salivary gland tumour. All mucoepidermoid carcinomas strongly express positive P63 [12]. The expression of the immunomarkers is not limited to an exact tumour type and the histopathological characteristics on H&E staining are the common standard for diagnosing tumours with slightly unclear histological features. In addition, distinctness of results globally may be associated with the technique sensitivity of IHC resulting in false negative or positive results.

## Conclusion

Pleomorphic adenoma is the most frequent tumour that originates from the parotid gland. Diagnosis may prove challenging in a setting of unusual findings, which can lead to blind alleys when recommending treatment. This was a rare case of pleomorphic adenoma that presented as a mere retroauricular mass. This case is an example that accurate history, diagnostic imaging, and satisfactory tissue sampling is crucial to exclude other lesions associated with the salivary glands. Appropriate diagnosis led to an excellent prognosis with less chance of recurrence.
